# Immersive virtual reality vs. traditional instruction for short-term learning and follow-up outcomes in the Bobath concept: a randomized controlled trial

**DOI:** 10.1186/s12909-026-09904-2

**Published:** 2026-08-20

**Authors:** Sena Cinarli, Gamze Aydogan, Ebru Sever, Gönül Ertunc Gulcelik, Halil Atmaca

**Affiliations:** 1 Department of Physiotherapy and Rehabilitation, Kocaeli Health and Technology University, Kocaeli, Turkey; 2 Institute of Health Sciences, Marmara University, Istanbul, Turkey; 3 Institute of Health Sciences, Istanbul Medipol University, Istanbul, Turkey; 4 Orthopedics and Traumatology, Istanbul, Turkey

**Keywords:** Virtual reality, Physiotherapy, Education, Clinical skills, Bobath Concept

## Abstract

**Background:**

Although virtual reality has increasingly been incorporated into health professions education, its effectiveness for teaching structured neurorehabilitation frameworks within physiotherapy remains insufficiently explored. Purpose: This study aimed to compare immersive virtual reality–based instruction with traditional education by evaluating theoretical knowledge and practical skills at short-term (2 days) and follow-up (2 weeks) assessments following Bobath concept instruction among physiotherapy students. Methods: In this assessor-blinded randomized controlled trial, 20 third-year physiotherapy students (mean age: 21.3 ± 1.1 years) were randomly assigned to immersive virtual reality–based instruction or traditional instruction. Both groups received standardized 45-minute training on postural control strategies within the Bobath concept. Learning outcomes were assessed using a multiple-choice theoretical test and a practical examination at 2 days (short-term) and 2 weeks (follow-up) after instruction. Results: Both groups showed a decrease in theoretical scores from the short-term to the follow-up assessment, with no significant between-group difference in change scores (*p* = 0.817). In contrast, between-group comparison of practical change scores showed a significant difference favoring the virtual reality group (*p* = 0.017, *r* = 0.54), with a median difference of + 15 points (95% CI: +5 to + 30). Within-group analyses indicated a significant decline in the traditional instruction group (*p* = 0.011, *r* = 0.81), whereas no significant change was observed in the virtual reality group (*p* = 0.666, *r* = 0.14). Practical scores increased by approximately 3% in the virtual reality group but decreased by 17% in the traditional instruction group from short-term to follow-up assessment. Conclusion: These findings suggest that immersive virtual reality may support the preservation of practical performance over time in physiotherapy education and may represent a promising instructional approach for teaching complex neurorehabilitation skills.

**Clinical trial number:**

ClinicalTrials.gov identifier: NCT07270107 (retrospectively registered on December 8, 2025).

## Introduction

Technological transformation in health professions education has progressively shifted instructional paradigms from passive, lecture-based knowledge transmission toward experiential and simulation-based learning models. Large-scale meta-analyses have demonstrated that simulation-based education consistently improves knowledge and skill acquisition compared with traditional instructional approaches [1, 2]. More recent systematic reviews further confirm the growing effectiveness of simulation and immersive technologies in enhancing learning outcomes in health professions training [3].

Within this pedagogical evolution, immersive virtual reality represents one of the most advanced forms of simulation-based learning, enabling learners to interact with dynamic three-dimensional environments through head-mounted displays that create a strong sense of presence and first-person engagement [4, 5]. Recent meta-analytic evidence suggests that immersive virtual reality can enhance clinical skill development and problem-solving performance in healthcare education compared with conventional instructional methods [6].

Unlike traditional didactic teaching, immersive virtual reality simultaneously engages visual, spatial, and motor systems, facilitating multisensory integration and embodied interaction. Recent neuroscience evidence indicates that multisensory learning promotes more efficient memory encoding and experience-dependent neural plasticity than unisensory learning [7]. From an embodied cognition perspective, clinical skill acquisition emerges through dynamic interactions between brain, body, and environment, whereby repeated sensorimotor engagement refines perception–action coupling and procedural memory formation [8]. These theoretical and neurobiological mechanisms provide a plausible rationale for the use of immersive virtual reality in the training of complex motor skills within health professions education.

Despite these theoretical advantages, the educational effectiveness of immersive virtual reality appears to be heterogeneous and outcome-dependent. A growing body of evidence demonstrates that virtual reality enhances student engagement, experiential learning, and, in some cases, knowledge retention [9, 10]. However, several randomized controlled trials report learning outcomes comparable to traditional teaching methods, with no statistically significant differences in core performance measures [11, 12]. Similarly, systematic reviews indicate that knowledge acquisition is often equivalent between immersive technologies and conventional educational approaches, despite consistent improvements in secondary outcomes such as engagement, satisfaction, and perceived learning experience [13].

Evidence synthesis studies further reinforce this mixed pattern of findings. One systematic review reported no statistically significant differences between virtual reality and high-fidelity simulation in 53.33% of studies [14], while another found that 60% of studies demonstrated comparable learning outcomes between immersive virtual reality and alternative instructional approaches [15]. These findings highlight considerable variability across instructional designs, outcome domains, and assessment time points. However, the maintenance of virtual reality–related learning outcomes remains insufficiently explored, as most studies focus primarily on short-term assessments, limiting conclusions regarding the persistence of knowledge and skill performance over time [16].

The Bobath concept is a widely used neurorehabilitation framework for individuals with neurological impairments and is commonly described as a problem-solving clinical approach focused on the assessment and facilitation of functional movement [17, 18]. Contemporary interpretations are grounded in systems-based models of motor control, motor learning, and neuroplasticity [19]. Effective application of the concept requires the development of tacit and kinaesthetic competencies that extend beyond declarative knowledge. In rehabilitation education, these competencies develop through repeated clinical interaction and are reflected in tactile clinical skills and embodied perception during therapeutic handling [20, 21]. Their acquisition relies on procedural memory formation in sensorimotor learning, whereby repeated practice induces plastic changes within distributed cortico-subcortical networks supporting the acquisition and consolidation of complex motor skills [22, 23]. These characteristics make the Bobath concept particularly challenging to teach through conventional instructional approaches and highlight the potential value of immersive simulation-based learning environments.

To date, few studies have systematically examined immersive virtual reality as an instructional modality for structured therapeutic frameworks within physiotherapy education. Although virtual reality has shown promise for improving engagement and psychomotor skill acquisition, its effectiveness for teaching complex neurorehabilitation approaches that depend heavily on kinaesthetic perception and therapeutic handling remains largely unexplored. Furthermore, previous studies rarely differentiate between theoretical knowledge acquisition and practical skill performance, despite these domains relying on distinct cognitive and sensorimotor processes that may respond differently to instructional approaches [24]. In particular, theoretical knowledge is primarily associated with cognitive processing and conceptual understanding, whereas practical skill performance depends on sensorimotor integration, procedural memory, and embodied interaction [25]. In addition, follow-up outcomes are seldom evaluated, even though short-term post-intervention performance may not accurately reflect the maintenance of learning over time [26]. Consequently, it remains unclear whether immersive virtual reality provides differential advantages across learning domains and time points, particularly in relation to the follow-up performance of clinically relevant sensorimotor competencies.

Therefore, the present assessor-blinded randomized controlled trial aimed to compare immersive virtual reality–based instruction with traditional teaching in the context of Bobath concept education for physiotherapy students. By distinguishing between theoretical knowledge and practical performance assessed through an objective structured clinical examination, and by evaluating these outcomes at short-term (2 days) and follow-up (2 weeks) assessments after instruction, this study aimed to compare the short-term and follow-up performance of students receiving immersive virtual reality–based or traditional instruction. It was hypothesized that immersive virtual reality–based instruction would result in superior theoretical knowledge and practical performance compared with traditional instruction.

## Methods

### Study design and participants

This parallel-group, assessor-blinded randomized controlled trial aimed to compare the effects of immersive virtual reality and traditional instruction on short-term learning and follow-up outcomes in the Bobath-related knowledge and skills in physiotherapy students. The study involved third-year undergraduate physiotherapy students during the spring semester. Ethical approval was obtained from the Non-Interventional Research Ethics Committee of Kocaeli Health and Technology University (Approval no: 26/11). All procedures conformed to the principles of the Declaration of Helsinki. Before participation, students were informed about the study objectives and procedures and provided written informed consent. Additional consent was obtained for video recording of assessment sessions, with assurances of anonymity and confidentiality. The study was designed and reported in accordance with the CONSORT 2010 guidelines [27]. Clinical Trial Registration: ClinicalTrials.gov (Identifier: NCT07270107), retrospectively registered and first posted on December 8, 2025. Data collection was conducted between October 20 and November 3, 2025.

As no directly comparable randomized controlled trials investigating immersive virtual reality-based instruction of the Bobath concept in physiotherapy education were available, a precise a priori sample size estimation based on directly comparable evidence was not feasible. However, effect sizes reported in the literature for virtual reality–based clinical skills training vary substantially. A recent systematic review and meta-analysis reported a large pooled effect size (Hedges’ g = 0.88, 95% CI: 0.47–1.29), indicating considerable variability across studies [28]. This variability makes it challenging to derive a precise a priori estimate for a context-specific intervention such as the present study. Therefore, a sensitivity analysis was performed to determine the minimum detectable between-group effect size with the available sample size.

A sensitivity power analysis was conducted using G*Power to determine the minimum detectable between-group effect size with the available sample size. With 10 participants per group, α = 0.05, power = 0.80, and a two-tailed independent-groups comparison, the minimum detectable effect size was approximately Cohen’s d = 1.32. This indicates that the study was sufficiently powered to detect only large between-group effects.

Eligible participants were third-year students taking the Neurophysiological Approaches II course for the first time. Inclusion criteria included no prior training in the Bobath concept and provision of written informed consent. Given that all participants had no prior formal training in the Bobath concept, baseline knowledge and skill levels were expected to be comparable across groups. In addition, baseline comparisons of demographic and academic characteristics, including academic performance, confirmed no statistically significant differences between groups. Students with conditions contraindicating virtual reality use, such as vestibular disorders, migraine, or epilepsy were excluded.

All eligible third-year physiotherapy students enrolled in the Neurophysiological Approaches II course (*n* = 60) were invited to participate through an open invitation. A convenience sampling approach based on voluntary participation was used. Participation was entirely voluntary, and no incentives were provided. Thirty students volunteered, of whom 20 met the inclusion criteria and were enrolled. Prior to randomization, participants completed the validated 10-item System Usability Scale (SUS) to assess baseline perceptions toward digital technologies. Academic performance was obtained from official university academic records and expressed as the cumulative academic grade average on a 0–100 scale. This variable was included solely to assess baseline comparability between the randomized groups.

Given that all participants had no prior formal training in the Bobath concept, baseline knowledge and skill levels were expected to be comparable across groups. In addition, baseline comparisons of demographic and academic characteristics, including academic performance, confirmed no statistically significant differences between groups.

**Fig. 1:**
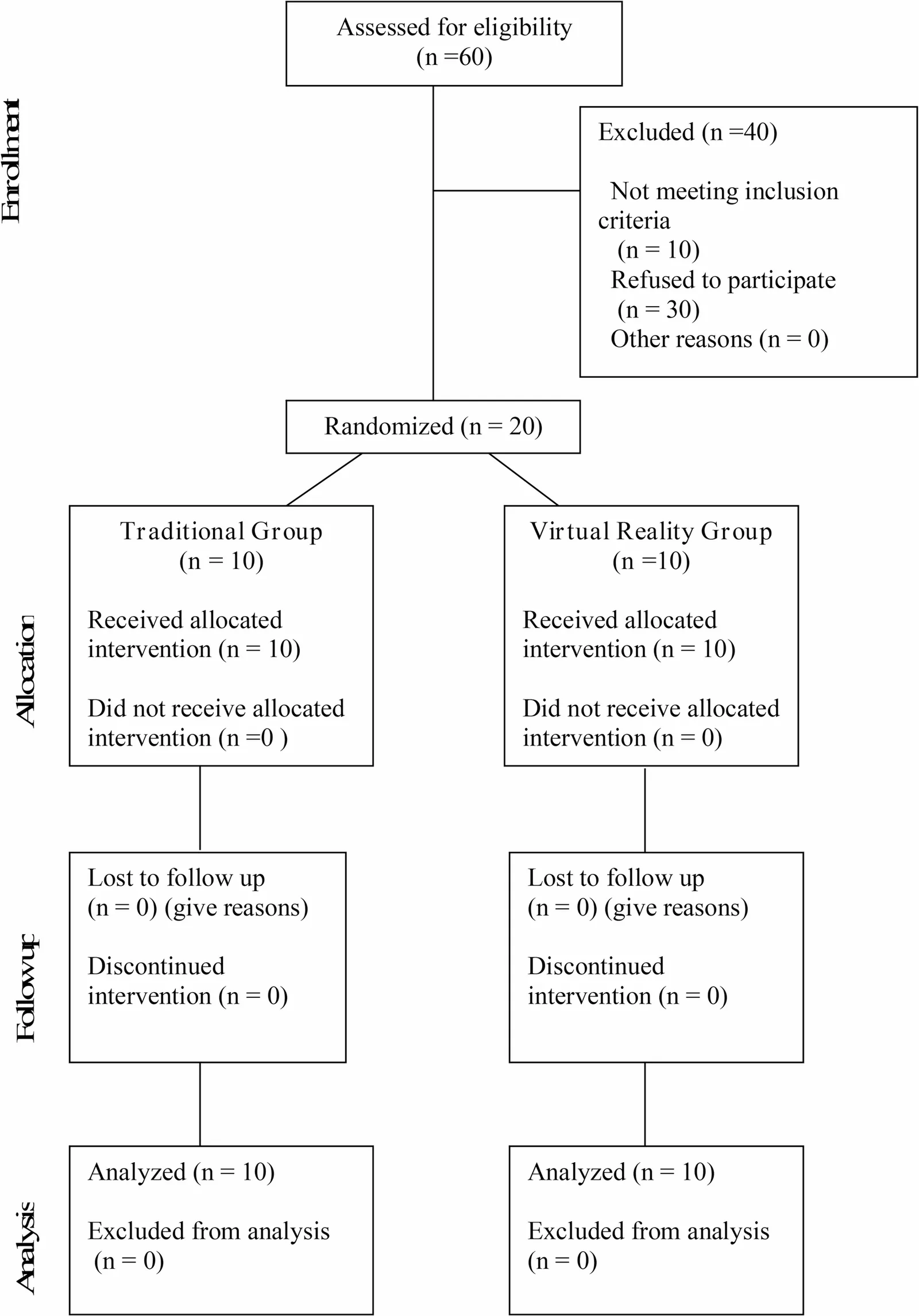
Flow chart of the study

Participants were randomly allocated to one of two groups using a computer-generated random allocation sequence prepared by an independent academic not involved in participant enrollment, instruction, or outcome assessment. The allocation sequence remained concealed from the investigators responsible for participant enrollment and outcome assessment until participant allocation had been completed. This was an assessor-blinded design in which two independent evaluators, blinded to both group allocation and assessment time point, independently scored all practical performance assessments. Both short-term and follow-up evaluations were video-recorded and independently scored using a standardized rubric (Fig. 1).

### Bobath concept intervention

Both groups participated in a single, standardized 45-minute instructional session on the Bobath concept, with a focus on postural control strategies that are fundamental to neurodevelopmental rehabilitation. All sessions were delivered by the same instructor, a physiotherapist holding a PhD in neurophysiological rehabilitation, to ensure consistency in content and delivery.

Training sessions were conducted in the same laboratory environment, on the same day but at different times for each group, eliminating the possibility of cross-group interaction. The instructional content was designed to address core principles of the Bobath concept, including the biomechanical foundations of postural alignment, facilitation of trunk control and symmetrical weight distribution, and the integration of sensory feedback to improve midline orientation and balance. Practical applications included guided postural adjustments during sitting, assisted transfers emphasizing active weight-bearing, and the use of tactile input to promote motor control and postural symmetry [29, 30]. The structure and duration of the training were intentionally aligned with typical academic time constraints, ensuring that both groups received equal exposure to the educational content. The instructional session delivered to the traditional group was recorded and used to create the 360° video content presented to the virtual reality group, ensuring that both groups received identical instructional material, with the only difference being the mode of delivery.

### Traditional education

Students in the traditional group participated in a live session consisting of a theoretical lecture followed by practical demonstrations of postural control strategies within parallel bars. The instructor promoted active engagement through note-taking and real-time questioning. After the session, students practiced the demonstrated techniques in small groups under supervision. Following the practice phase, a brief debriefing was conducted to reinforce key clinical concepts.

### Virtual reality education

**Fig. 2:**
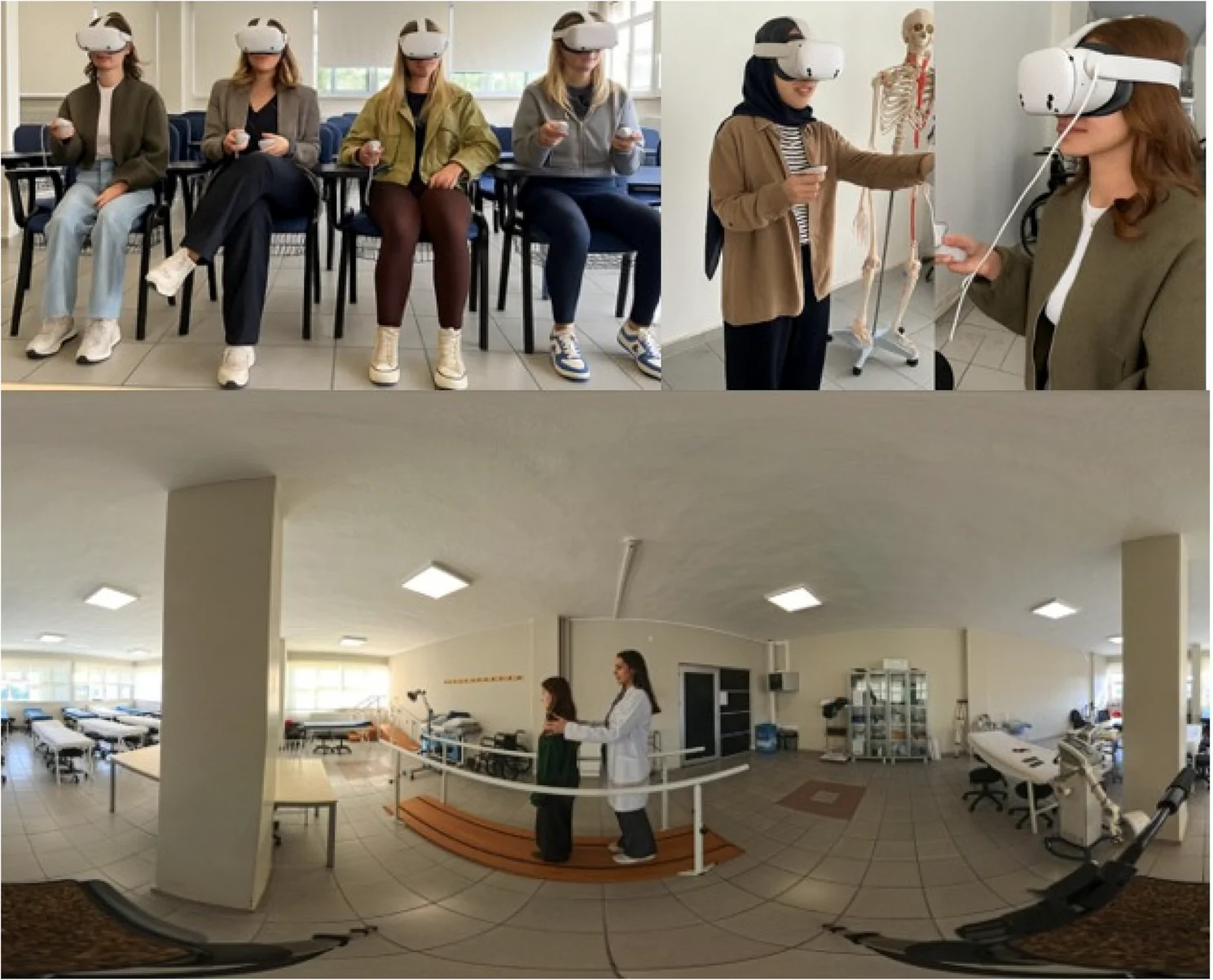
Immersive virtual reality–based Bobath concept instruction. Top panel: Students simultaneously viewing pre-recorded immersive 360° instructional videos using head-mounted displays. Bottom panel: Supervised hands-on practice performed after completion of the VR viewing session to reinforce the observed Bobath facilitation techniques

Students in the virtual reality group experienced the Bobath training content through immersive 360° video delivered via head-mounted virtual reality displays. The instructional material included theoretical narration and live demonstrations of facilitation techniques performed on a volunteer within a simulated clinical environment. During the session, students viewed the immersive content simultaneously in a classroom setting using individual VR headsets (Fig. 2, top panel). Following completion of the immersive 360° video viewing session, students participated in supervised hands-on practice outside the virtual environment to reinforce the observed facilitation techniques. This practice consisted of therapist–participant simulation under instructor supervision and was designed to strengthen kinaesthetic understanding and procedural application of Bobath-based postural control strategies (Fig. 2, bottom panels). After completion of the practice phase, a brief debriefing session was conducted to encourage reflection and clarify key clinical concepts.

### Outcome measures

Primary outcomes included theoretical knowledge of postural strategies and practical performance in their application. Assessments were conducted at two points: two days after instruction (short-term) and two weeks later (follow-up). A 10-item multiple-choice test assessed theoretical knowledge, focusing on principles and clinical applications of the Bobath concept related to postural control. The test was designed to provide a standardized assessment of core theoretical knowledge and fundamental clinical applications rather than a comprehensive evaluation of all theoretical competencies. The multiple-choice format was selected to ensure objective scoring with minimal examiner subjectivity, whereas practical application of the Bobath concept was evaluated separately through the OSCE [31]. Each correct response was awarded one point, and total scores were converted to a 0–100 scale through a linear transformation solely to facilitate comparison with the practical examination scores. The same test was used at both assessment time points.

Practical performance was evaluated through an objective structured clinical examination (OSCE) composed of three stations addressing different aspects of Bobath-based postural strategies. The OSCE stations were designed to assess patient positioning, postural alignment, facilitation techniques, and weight transfer strategies related to Bobath-based postural control. Each station was scored using a standardized multi-item 3-point checklist (0 = not performed, 1 = partially performed, 2 = correctly performed), and total scores were converted to a 0–100 scale [32, 33]. The OSCE stations were designed to assess the application of Bobath-related postural control strategies, including patient positioning, facilitation techniques, postural alignment, and weight transfer during functional tasks. Students were randomly paired and individually assessed under blinded conditions. Performances were video-recorded, anonymized, and coded prior to evaluation to prevent identification of group allocation or assessment sequence. The recordings were independently scored by two evaluators using a standardized rubric, and both evaluators remained blinded to group allocation and assessment time point throughout the scoring process. The second, unannounced assessment replicated the same procedures to evaluate follow-up performance of both theoretical and practical skills. Inter-rater reliability for the OSCE ratings was assessed using the intraclass correlation coefficient (ICC; two-way random-effects model, absolute agreement, single measures) and was excellent (ICC > 0.95).

### Statistical analysis

All statistical analyses were conducted using IBM SPSS Statistics (Version 23, NY, USA). Data distribution was assessed using the Shapiro–Wilk test. Baseline comparisons of demographic and background characteristics between groups were performed using independent-samples t-tests for normally distributed continuous variables and Fisher’s exact test for categorical variables. In contrast, outcome variables were analyzed using nonparametric methods due to the small sample size and non-normal distribution of several measures. Within-group changes between short-term and follow-up assessments were evaluated using the Wilcoxon signed-rank test for both the virtual reality and traditional instruction groups. Between-group differences in change over time were assessed using the Mann–Whitney U test applied to individual change scores (Δ = follow-up – short term). Bootstrap percentile 95% confidence intervals were calculated for the between-group differences in median change scores. To provide a robust representation of change, median delta values (median of individual raw differences) were calculated and reported alongside interquartile ranges. Additionally, mean percentage changes were computed descriptively as the average of individual relative changes [(follow-up – short term) / short term × 100] to illustrate the overall magnitude and direction of change across participants. While mean-based percentage changes provide contextual interpretation, statistical inference was based on nonparametric comparisons of individual change scores. Effect sizes were calculated as r = Z/√N and interpreted according to conventional thresholds (0.10 = small, 0.30 = moderate, 0.50 = large). Statistical significance was set at *p* < 0.05.

## Results

A total of 20 female third-year undergraduate physiotherapy students (mean age: 21.3 ± 1.1 years) participated in the study. No statistically significant differences were observed between the virtual reality and traditional instruction groups regarding age (*p* = 0.54), prior virtual reality experience (*p* = 0.65), system usability scale scores (*p* = 0.72), or academic performance (*p* = 0.06), indicating no baseline differences between groups (Table 1).

**Table 1:** Baseline demographic and background characteristics of participants (*n* = 20)*

Groups	Age (years)	Previous virtual reality experience (Yes/No)	System usability scale score	Academic performance (0-100)
Virtual reality (*n* = 10)	21.5 ± 1.2	4 / 6	96.9 ± 1.7	86.5 ± 2.4
Traditional (*n* = 10)	21.1 ± 1.0	3 / 7	97.3 ± 2.0	84.5 ± 1.6
p value	0.54	0.65	0.72	0.06

*Continuous variables are presented as mean ± standard deviation. Categorical variables are presented as counts. Between-group comparisons were performed using independent-samples t-tests for continuous variables and Fisher’s exact test for categorical variables

In the theoretical examination, both groups demonstrated a decline from the short-term to the follow-up assessment. The median change was − 15 (− 20 to 0) in the virtual reality group and − 10 (− 30 to 0) in the traditional instruction group. However, the between-group comparison of change scores revealed no significant difference (*p* = 0.817, *r* = 0.06). The median between-group difference in theoretical change scores was − 5 points (bootstrap 95% CI: −20 to + 25).

In contrast, the practical examination showed divergent performance trajectories between groups. The traditional instruction group exhibited a decline in performance with a median change of − 15 (− 20 to − 10), whereas the virtual reality group showed no significant change, with a median change of 0 (− 10 to 10). Between-group comparison of individual change scores demonstrated a significant difference favoring the virtual reality group (*p* = 0.017, *r* = 0.54), indicating a moderate-to-large effect size. The median between-group difference in practical examination change scores was + 15 points (bootstrap 95% CI: +5 to + 30) (Table 2).

**Table 2:** Short-term and follow-up examination scores and change values (Δ) in the virtual reality and traditional instruction groups (*n* = 20)*

Outcome	Group	Short-term	Follow-up	Median Delta	*p*	ES
Theoretical	Virtual reality	80 (70–90)	70 (70–80)	−15 (− 20–0)	0.817	0.06
	Traditional	85 (80–100)	70 (60–80)	−10 (− 30–0)		
Practical	Virtual reality	95 (80–100)	90 (80–100)	0 (− 10–10)	0.017	0.54
	Traditional	90 (80–100)	75 (70–80)	−15 (− 20 – −10)		

*Data are presented as median (P25–P75). p values represent between-group comparisons of individual change scores (Δ = follow-up – short-term) using the Mann–Whitney U test; ES represents effect size r (Z/√N). A positive Δ value indicates improvement, whereas a negative value indicates decline from short-term to follow-up assessment

**Fig. 3:**
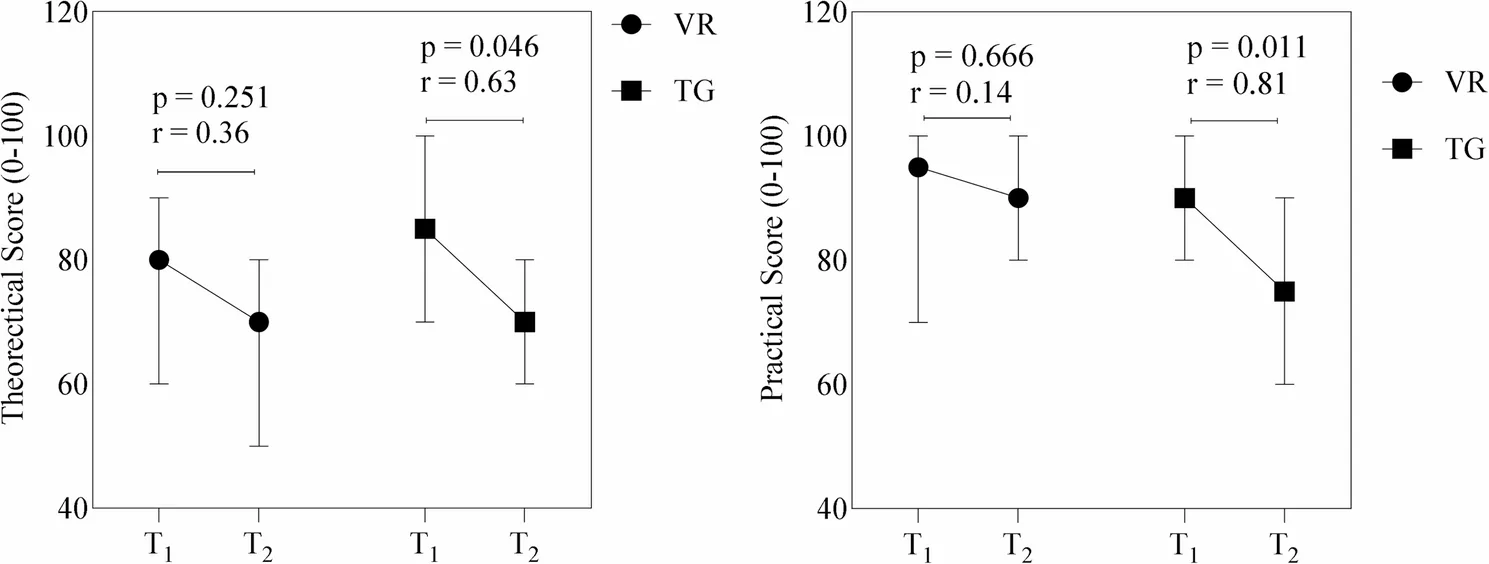
Within-group changes in theoretical and practical examination scores from short-term to follow-up assessment in the virtual reality and traditional instruction groups. Data are presented as median (P_25_-P_75_)

Within-group analyses revealed a significant decline in the traditional instruction group for both the theoretical (*p* = 0.046, *r* = 0.63) and practical examinations (*p* = 0.011, *r* = 0.81), representing large effect sizes, from short-term to follow-up assessment. In contrast, no significant change was observed in the virtual reality group for either the theoretical (*p* = 0.251, *r* = 0.36) or practical outcomes (*p* = 0.666, *r* = 0.14) (Fig. 3).

**Fig. 4:**
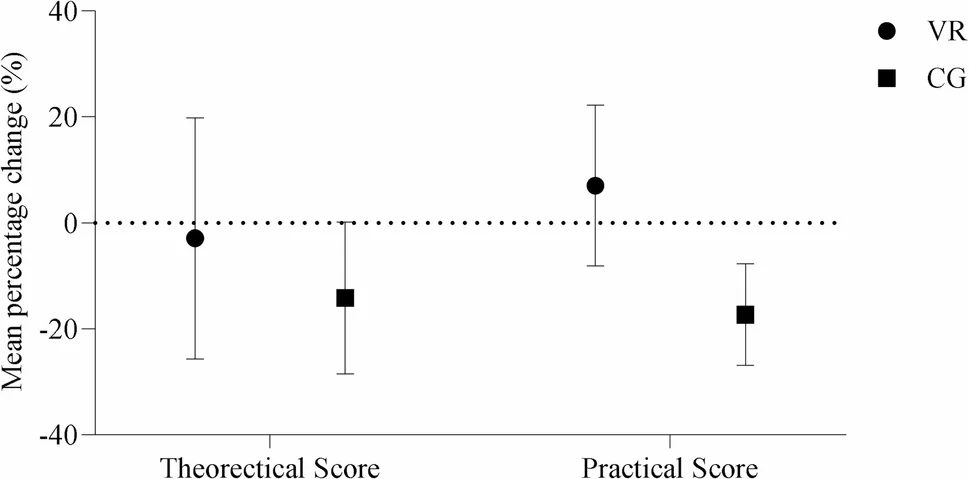
Mean percentage change in theoretical and practical examination scores from short-term to follow-up assessment in the virtual reality and traditional instruction groups

The virtual reality group demonstrated relatively stable performance over time, with a mean percentage change of approximately − 7% in the theoretical examination and + 3% in the practical assessment from short-term to follow-up measurement. In contrast, the traditional instruction group exhibited a larger decline in both domains, with mean percentage changes of approximately − 14% in the theoretical examination and − 17% in the practical assessment (Fig. 4). These percentages were calculated as the average of individual relative changes and are presented descriptively.

## Discussion

This randomized controlled trial compared immersive virtual reality–based instruction with conventional education for teaching the Bobath concept in physiotherapy students. Both instructional approaches were evaluated at 2 days (short-term) and 2 weeks (follow-up) post-training. While no differences were observed between groups in either theoretical or practical performance the short-term assessment, performance trajectories differed for practical skills at follow-up. The traditional instruction group demonstrated a decline in practical performance over time, whereas the virtual reality group exhibited relatively stable performance. Consequently, between-group comparison of change scores revealed a statistically significant advantage for the virtual reality group, indicating better preservation of practical performance across the assessment interval.

These findings extend the limited body of research examining the use of virtual reality in physiotherapy education beyond traditional anatomy instruction [34]. Although immersive virtual reality has gained recognition across various health disciplines, its pedagogical integration within physiotherapy and rehabilitation training remains at an early stage [35, 36]. In dental education, Huang et al. reported superior practical performance with a VR-based simulation compared with conventional instruction, although retention was not evaluated [37]. Similarly, Lee et al. demonstrated improved short-term theoretical knowledge and procedural confidence following VR-based blood transfusion training in nursing students [38].

Ekstrand et al. conducted a randomized, blinded trial comparing virtual reality-based and traditional instruction for teaching spatial neuroanatomy to medical students. After training, participants completed both immediate and one-week retention tests, and the virtual reality group achieved significantly higher procedural scores, demonstrating performance at follow-up. The study shared methodological similarities with the present design, including blinded assessment and delayed follow-up testing, although its primary focus was spatial and anatomical comprehension rather than psychomotor skill acquisition in physiotherapy practice [39]. Similarly, Baratz et al. reported sustained improvements in cognitive and procedural performance following mixed-reality cadaveric training [40]. In line with these findings, the current study reinforces the pedagogical value of immersive virtual reality for procedural learning, while extending its application to physiotherapy-specific motor skill acquisition.

The use of realistic, embodied interaction in virtual reality may represent a crucial step toward bridging theoretical instruction and clinical skill performance within rehabilitation education. Consistent with the present findings, randomized and simulation-based studies have demonstrated that immersive virtual reality and related technologies can enhance clinical skill development, clinical reasoning, manual skill performance, competency acquisition, knowledge retention, and learner engagement in health professions education [41–45]. Collectively, these findings support the growing pedagogical role of immersive virtual reality as a complementary educational approach for promoting both cognitive and psychomotor learning.

Yet, the literature offers limited consensus on the optimal timeframe for assessing knowledge and skill performance over time. The present study adopted a two-week follow-up interval, consistent with previous research evaluating short- to medium-term retention in educational and motor learning contexts [46, 47], balancing ecological validity with practical feasibility within an academic setting. From a neurophysiological perspective, retention intervals spanning days to weeks are commonly used to capture early consolidation processes, during which motor memory becomes progressively stabilized and resistant to interference [48]. In this regard, a two-week interval represents a plausible and methodologically appropriate timeframe for detecting early retention of newly acquired skills, as retention assessments at approximately two weeks have been commonly employed in procedural skill learning studies, with evidence demonstrating comparable performance outcomes at this time point across different instructional modalities [49]. However, it should be acknowledged that this timeframe primarily reflects short-term or early consolidation processes and may not be sufficient to capture the long-term retention patterns, as longer follow-up periods are often recommended to assess sustained retention over time.

The more favorable pattern of change observed in practical performance over time in the virtual reality group may be interpreted within a theoretical framework of immersive learning. Rather than reflecting superior performance at a single time point, this pattern suggests a relative preservation of skill execution across the assessment interval. Although neurophysiological mechanisms were not directly assessed in the present study, immersive environments may engage multisensory and embodied learning processes that are relevant to procedural skill acquisition. In this context, active engagement, emotional salience, visual–spatial input, and kinesthetic observation may provide a plausible framework for understanding the observed pattern of practical performance retention [50]. Emerging evidence from virtual reality–based rehabilitation research has also highlighted the potential role of visual attention processes during task performance in virtual environments. For example, Maldonado Díaz et al. reported increased fixation stability and changes in gaze behavior during virtual reality–based balance training, suggesting a more efficient allocation of attention with repeated task exposure [51]. Although visual attention was not directly assessed in the present study, these findings support the potential relevance of perceptual and visuomotor processes within virtual reality–based learning and rehabilitation environments. These interpretations are consistent with motor learning principles in rehabilitation, which emphasize repetitive, task-specific practice and enriched sensory feedback as factors that may support the acquisition and retention of motor skills [52, 53].

In contrast, previous research indicates that although immersive virtual reality environments often enhance engagement, motivation, and subjective learning experiences, these effects do not consistently translate into improved immediate or short-term learning outcomes [54]. Such effects have been partly attributed to a novelty effect, which may diminish with repeated exposure and does not necessarily result in superior knowledge or skill acquisition [55], although some evidence suggests that engagement may be sustained over time [56]. This distinction between initial engagement and deeper learning processes may help explain why no clear advantage was observed in short-term outcomes, while a more favorable pattern of performance change over time emerged in the virtual reality group [57].

It should also be noted that the immersive virtual reality approach used in the present study was based on 360° video content, which differs from fully interactive virtual reality simulations. While 360° video provides a high level of visual immersion and supports observational learning, it does not allow users to actively manipulate the environment or engage in real-time interaction with virtual elements. From a motor learning perspective, this distinction is important. Fully interactive virtual reality environments may facilitate active motor engagement, error-based learning, and sensorimotor adaptation through user-driven interaction. In contrast, 360° video–based virtual reality primarily supports observational learning and cognitive processing of movement strategies rather than active motor execution. Therefore, the present findings should be interpreted within the context of this specific type of immersive but non-interactive virtual reality, which primarily emphasizes observational learning rather than active motor engagement.

Notably, no significant between-group differences were observed in theoretical knowledge outcomes at either the short-term or follow-up assessments. In addition, both groups demonstrated a decline in theoretical performance over time, suggesting that neither instructional approach was sufficient to maintain knowledge across the assessment interval. This finding is consistent with prior randomized controlled studies indicating that immersive virtual reality does not consistently outperform traditional instructional approaches in cognitive domains. For instance, a randomized trial in obstetrics education reported a higher proportion of correct responses in the virtual reality group compared with traditional 2D instruction (70% vs. 56%), although this difference did not reach statistical significance [58]. Comparable findings have also been reported in nursing education, where no significant differences in knowledge outcomes were observed between 360° virtual reality video and face-to-face demonstration (*p* = 0.952), despite the immersive nature of the intervention.

One possible explanation is that theoretical learning primarily depends on cognitive processing and information recall, which can be effectively supported by both conventional and technology-enhanced instructional methods. However, immersive virtual environments may introduce additional cognitive demands due to the need to process multisensory inputs and navigate within the environment. Previous research highlights that the visual complexity and high degree of freedom in virtual environments may lead to information overload and increased mental effort, thereby elevating extraneous cognitive load [59]. Notably, previous randomized evidence indicates that more than half of participants (62.5%) experienced virtual reality–related symptoms, including discomfort and visual strain, which may have negatively influenced cognitive processing during learning [60]. This increased cognitive load may reduce the efficiency of information encoding and limit knowledge acquisition. In support of this, studies have shown that mental effort and interface-related demands in immersive environments may negatively predict cognitive learning outcomes, particularly knowledge test performance [61].

In addition, immersive virtual reality is not without practical and physiological limitations. Cybersickness is a well-documented constraint of virtual reality–based learning environments; for example, approximately 47% of undergraduate participants in a virtual reality–based classroom reported symptoms such as dizziness and discomfort [62]. Common symptoms include nausea, disorientation, and visual fatigue, which may negatively affect learner comfort and engagement [63]. Furthermore, the need for specialized equipment and technical infrastructure may influence the feasibility of implementation, particularly in resource-limited educational settings. In contrast, traditional instructional methods are typically more accessible, require fewer technological resources, and may impose lower cognitive and perceptual demands on learners. This may partly explain why comparable knowledge outcomes were observed between instructional modalities in the present study. Therefore, rather than replacing conventional teaching approaches, immersive virtual reality may be more appropriately positioned as a complementary tool within blended learning models [64], particularly for domains requiring experiential and sensorimotor learning.

To the best of current knowledge, this study is the first randomized controlled trial to compare immersive virtual reality–based instruction with traditional instruction for teaching the Bobath concept in physiotherapy education. A key strength lies in the randomized controlled design and use of blinded raters, which minimized bias and enhanced internal validity. The two evaluation points enabled assessment of performance at short-term and follow-up time points.

### Limitations

Despite these strengths, certain limitations should be noted. The study was conducted at a single institution, and participation was voluntary, introducing the possibility of self-selection bias. Students who chose to volunteer may have differed from those who did not volunteer with respect to motivation, interest in educational innovation, or learning engagement, which may limit the generalizability of the findings to the broader population of physiotherapy students. Furthermore, the single-institution setting limits external validity, and larger multicenter studies would strengthen the generalizability of future findings. The immersive virtual reality intervention used in this study was based on 360° video content rather than fully interactive virtual reality, which should be considered when interpreting the findings. Consequently, the results may not be directly generalizable to immersive systems involving active user interaction, real-time manipulation, or adaptive motor engagement. Accordingly, the conclusions of the present study should be interpreted within the context of this specific type of immersive observational learning environment. Furthermore, cybersickness symptoms or headset-related discomfort were not formally assessed using a standardized instrument, which may limit interpretation of participant experiences during immersive virtual reality exposure. All participants in the present study were female. Although evidence regarding gender-related differences in immersive virtual reality learning outcomes remains inconclusive, the use of a single-gender sample may limit the generalizability of the findings to male students and more gender-diverse populations [65]. The absence of a pre-intervention assessment may limit the ability to directly confirm baseline equivalence in domain-specific knowledge and skills, particularly regarding the interpretation of practical performance outcomes. However, comparable academic performance and the lack of prior exposure to the Bobath concept reduce the likelihood of substantial baseline differences between groups. In addition, independent study behaviors during the interval between the intervention and follow-up assessments were not objectively monitored. Although no additional Bobath instruction or structured practice was provided by the research team during this period, differences in self-directed learning outside the study cannot be completely excluded and may have influenced the observed outcomes. Furthermore, the repeated administration of the same theoretical test at both assessment time points may have introduced potential recall or test familiarity effects, although theoretical scores did not improve over time in either group. The relatively small sample size should also be considered when interpreting the findings. Sensitivity analysis indicated that, with 10 participants per group, the study was powered to detect only large between-group effects; therefore, smaller or more subtle differences, particularly in theoretical knowledge outcomes, may not have been detected. Moreover, the retrospective registration of the trial should be considered a methodological limitation related to transparency and trial reporting, although all study procedures were conducted according to predefined protocols and ethical approval requirements. Finally, incorporating qualitative feedback, such as interviews or focus groups, could further enrich understanding of student experiences and engagement.

## Conclusion

This study provides preliminary evidence that immersive virtual reality can support instruction of the Bobath concept in physiotherapy education. While both traditional and virtual reality-based teaching produced comparable short-term performance, the virtual reality group demonstrated a more favorable pattern of practical performance preservation at follow-up. These results highlight the potential of immersive 360° virtual reality to support the maintenance of procedural performance following instruction in physiotherapy education. However, the findings should be interpreted within the context of a non-interactive immersive learning environment and a relatively short follow-up period. Future research should aim to replicate these findings in larger and more diverse populations, incorporate long-term follow-up assessments, and explore strategies for optimal integration of immersive virtual reality into health sciences curricula to maximize its educational and clinical impact.

## Data Availability

The data that support the findings of this study are available from the corresponding author upon reasonable request.The data that support the findings of this study are available from the corresponding author upon reasonable request. The data are not publicly available due to privacy and ethical restrictions.
